# HPT Axis Dysregulation in Mood and Anxiety Disorders: The Clinical Utility of Routine Hormonal Dosing in Psychiatric In-Patients

**DOI:** 10.3390/diseases14060211

**Published:** 2026-06-11

**Authors:** Georgiana-Adriana Toma, Elena Coman, Antonia Ioana Vasile, Simona Trifu

**Affiliations:** 1 Department of General Medicine, “Carol Davila” University of Medicine and Pharmacy, 041914 Bucharest, Romania; 2 Doctoral School, “Carol Davila” University of Medicine and Pharmacy, 041914 Bucharest, Romania; 3 Department of Clinical Neurosciences, “Carol Davila” University of Medicine and Pharmacy, 041914 Bucharest, Romania

**Keywords:** thyroid dysfunction and mood disorders, major depressive disorder and TSH, bipolar disorder thyroid hormones, subclinical hypothyroidism depression, non-thyroidal illness psychiatric in-patients, FT4 FT3 correlations in affective disorders

## Abstract

Background/Objectives: Thyroid dysfunction is frequently associated with mood and anxiety disorders, yet the directionality and diagnostic specificity of this relationship remain debated. Although numerous studies have examined major depressive disorder (MDD) and bipolar disorder (BD) separately, comparative data including generalized anxiety disorder (GAD) and accounting for non-thyroidal illness (NTI) effects remain scarce. This study aimed to evaluate thyroid function and its correlation with affective symptom severity across MDD, GAD, and BD in an inpatient cohort. Methods: Eighty-eight hospitalized patients with MDD, GAD, or BD were included in the study (MDD = 30, GAD = 30, BD = 28). Serum levels of TSH, FT4, and FT3 were measured 24–48 h after admission to minimize the influence of NTI. Psychiatric assessment included the Montgomery-Åsberg Depression Rating Scale (MADRS), Hamilton Anxiety Scale (HAM-A), and Young Mania Rating Scale (YMRS). Between-group differences were analyzed using ANOVA, and associations between thyroid parameters and symptom severity were examined using correlation and regression analyses. Results: ANOVA revealed that patients with MDD had significantly higher TSH levels compared with those with GAD and BD (*p* < 0.01). MDD patients also showed a higher prevalence of subclinical hypothyroidism (33.3%) than patients with GAD (13.3%) and BD (7.1%), as well as a higher prevalence of overt hypothyroidism (13.3%) compared with GAD (0%) and BD (7.1%). TSH levels correlated positively with MADRS scores (r = 0.45, *p* < 0.05) and HAM-A scores (r = 0.38, *p* < 0.05), particularly within the MDD group. In BD, FT4 and FT3 levels were elevated and positively correlated with YMRS scores (FT4: r = 0.30, *p* < 0.05; FT3: r = 0.42, *p* < 0.05). In regression analysis within the MDD subgroup, both hypothyroidism and male sex were independently associated with higher MADRS scores, indicating greater depressive symptom severity. Conclusions: These findings suggest diagnosis-specific patterns of thyroid dysfunction among psychiatric inpatients. Higher TSH levels and increased rates of hypothyroidism were most prominent in MDD and were associated with greater depressive and anxiety symptom severity, whereas elevated FT4 and FT3 levels in BD were associated with manic symptom severity. The results support systematic thyroid screening in depressive admissions, hormone-informed monitoring in bipolar disorder, and a more integrated endocrine–psychiatric approach to clinical care.

## 1. Introduction

Numerous studies over the years have examined in detail the connection between abnormal thyroid function and psychiatric manifestations. Although numerous studies have explored thyroid dysfunction in isolated psychiatric conditions such as major depressive disorder (MDD) or bipolar disorder (BD), comparative analyses that simultaneously evaluate multiple affective diagnoses—including generalized anxiety disorder (GAD)—remain limited. Existing literature is characterized by substantial heterogeneity in methodology, patient populations, and endocrine markers assessed, which limits the generalizability of findings and underscores the need for more clinically homogeneous inpatient-based investigations. Most prior studies have been conducted in outpatient or population-based samples, whereas data derived from acutely hospitalized psychiatric patients—who often present more severe symptomatology and complex biological alterations—remain scarce. Importantly, few studies systematically account for the confounding effect of non-thyroidal illness (NTI), which may significantly distort thyroid hormone interpretation in acute psychiatric settings. Despite extensive research, the clinical utility of routine thyroid hormone assessment in psychiatric practice remains insufficiently defined, particularly in relation to symptom severity and diagnostic differentiation. Thyroid dysfunction can lead to a wide range of neuropsychiatric symptoms, including depression, dementia, manic episodes, and Hashimoto’s autoimmune encephalopathy [1].

The relationship between the thyroid and BD is complex, with hormonal changes influencing both the severity and type of affective episodes. A recent study (2023) identified clinical signs of thyroid dysfunction in 27% of patients with a first episode of mania/hypomania and in 60% of those with bipolar depressive episodes [2].

Recognition of this type of interaction is important for proper patient assessment and for the formulation of personalized therapeutic approaches. Correction of thyroid imbalances has been shown to be useful as an adjunctive therapy in the treatment of affective disorders, contributing to the improvement of symptoms and increasing the effectiveness of antidepressant medication [3]. Studies conducted over the past three decades have consistently supported this hypothesis, suggesting a possible interaction between the thyroid and monoaminergic systems as a biological mechanism of action [4].

A key element in this equation is the existence of a bidirectional relationship: not only can thyroid dysfunction cause or worsen psychiatric symptoms, but psychiatric disorders can also negatively influence thyroid function [5]. Psychiatric disorders, particularly depression and anxiety, are associated with chronic activation of the stress response system, leading to dysregulation of the hypothalamic–pituitary–thyroid (HPT) axis. Elevated cortisol levels may suppress hypothalamic TRH secretion and alter peripheral thyroid hormone metabolism. Alterations in monoaminergic neurotransmission (e.g., serotonin and dopamine), which are central to affective disorders, can interfere with thyroid hormone regulation and feedback mechanisms. Psychiatric disorders are increasingly linked to low-grade systemic inflammation, which may influence deiodinase activity and promote patterns consistent with non-thyroidal illness, such as reduced FT3 levels. Additionally, factors such as poor nutrition, sleep disturbances, and psychotropic medications (e.g., lithium, antidepressants) can further disrupt thyroid function.

**Figure 1:**
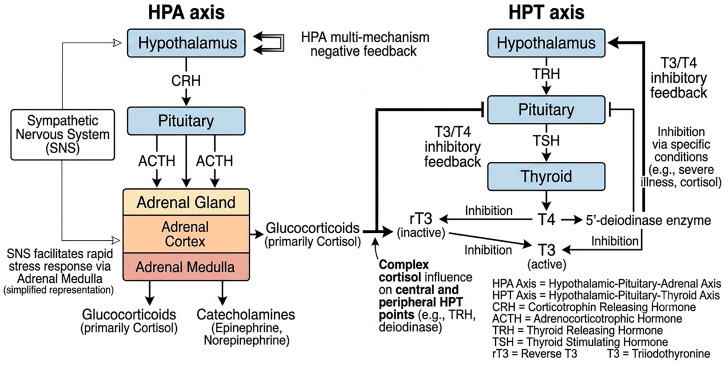
Biological mechanisms underlying the bidirectional relationship between thyroid function and psychiatric disorders.

The relationship between thyroid function and psychiatric disorders is bidirectional and mediated by complex neuroendocrine, immune, and metabolic mechanisms. On one hand, thyroid hormones play a crucial role in regulating central nervous system activity, including modulation of monoaminergic neurotransmission (serotonin, dopamine, noradrenaline), cerebral metabolism, and synaptic plasticity, thereby influencing mood, cognition, and behavior. On the other hand, psychiatric disorders—particularly those associated with chronic stress—can disrupt the hypothalamic–pituitary–thyroid (HPT) axis through several pathways. Persistent activation of the hypothalamic–pituitary–adrenal (HPA) axis leads to elevated cortisol levels, which may suppress hypothalamic thyrotropin-releasing hormone (TRH) and pituitary thyroid-stimulating hormone (TSH) secretion, as well as alter peripheral conversion of thyroxine (T4) to triiodothyronine (T3). In parallel, inflammatory processes frequently observed in affective disorders, characterized by increased proinflammatory cytokines (e.g., IL-6, TNF-α), can interfere with deiodinase activity and contribute to patterns consistent with NTI. Additionally, behavioral factors (such as sleep disturbances and nutritional deficits) and psychotropic medications (e.g., lithium) may further influence thyroid function. These mechanisms suggest that thyroid alterations observed in psychiatric patients may represent both contributing factors and adaptive responses to the underlying psychopathology (Figure 1).

In addition, the concept of non-thyroid disease (NTD), frequently encountered in psychiatric inpatients, should be mentioned. NTD involves changes in thyroid hormone levels that do not signal a thyroid pathology per se, but reflect a secondary reaction to a systemic disease or a severe psychiatric disorder [6]. In this context, some authors described how immune dysregulation following viral infection may trigger organ-specific autoimmune phenomena, supporting the broader concept that systemic immune–endocrine interactions—such as those underlying thyroid axis instability—can contribute to affective dysregulation [7]. These changes may vary depending on the type and severity of the psychiatric condition, and the intensity of the observed hormonal imbalances is often proportional to the severity of the psychiatric symptomatology.

In addition to overt thyroid dysfunction, which is widely recognized in the medical literature, a growing body of research indicates that subclinical changes in thyroid function can also have a significant impact on mental health [1]. Thus, subclinical hypothyroidism, characterised by normal FT4 values and a slight increase in TSH (above 4.5 mIU/L, but below 10 mIU/L), has been associated with the development of depressive symptoms [8]. Similarly, when TSH is low (below 0.4 mIU/L), but FT4 and FT3 levels remain within normal limits, subclinical hyperthyroidism is defined, which can also influence the patient’s mental state [1].

Although the theoretical background of this work recognizes the multifactorial role of the endocrine system in affective disorders, including the HPA and HPG axes, the present study focused exclusively on the analysis of the hypothalamic–pituitary–thyroid (HPT) axis. This methodological decision was based on the high prevalence of thyroid dysfunctions in the psychiatric population, their direct and often reversible clinical impact, and the need for an in-depth analysis of thyroid markers as potential biomarkers of status and severity.

Having in mind the gaps in existing literature, our study had two main objectives. The first aim was to evaluate thyroid function or dysfunction among several inpatients with affective and anxiolytic disorders (MDD, GAD, and BD). The second aim was to assess whether variations in thyroid functional status were related to illness severity, as measured by standardized psychiatric clinical rating scales. Our aims highlighted the role of thyroid function in the etiopathogenesis of affective disorders and its prognostic potential, supporting the systematic integration of endocrine assessment into psychiatric practice. In this way, it aims to outline an interdisciplinary perspective that can contribute to a more personalized and effective therapeutic approach to the psychiatric patient.

## 2. Materials and Methods

### 2.1. Study Design and Objectives

Our study had an observational, cross-sectional design, which allowed simultaneous assessment of thyroid hormone levels and the severity of psychiatric symptoms at a single point in time, in a hospital setting.

The specific objectives of our study were as follows:

- To characterize the thyroid hormonal profiles of psychiatric inpatients diagnosed with MDD, GAD, and BD;
- To characterize the affective and clinical severity profile of these patients using standardised psychiatric rating scales;
- To examine the associations between thyroid function parameters and psychiatric symptom severity;
- To compare thyroid function parameters and psychiatric scale scores between diagnostic groups;
- To estimate the prevalence of thyroid dysfunction (euthyroidism, subclinical/overt hypothyroidism, subclinical/overt hyperthyroidism, non-thyroid disease) in the overall sample and across diagnostic groups;
- To assess the extent to which thyroid function may predict disorder-specific clinical severity scores, after adjusting for sociodemographic and clinical covariates.

### 2.2. Study Population

The study was conducted among a cohort of 88 inpatients hospitalized at the Clinical Hospital of Psychiatry of Bucharest, within a period of 2 years. Their age varied between 18 and 65 years old (with a mean of 42.5 years and a standard deviation of ±12.3 years).

These patients had the primary diagnosis of MDD, GAD, or BP (comprising manic episodes), which was established at hospital admission by the admitting psychiatrist, according to DSM-5 diagnostic criteria. The diagnosis was based on a semi-structured clinical psychiatric interview, complemented by clinical observation and available medical records. When comorbid or overlapping affective and anxiety symptoms were present, patients were classified according to the primary diagnosis judged to account for the main reason for admission and the predominant clinical presentation.

All patients included in the study were admitted to a psychiatric department for the first time.

Among patients diagnosed with MDD, the depressive episode was classified as major in severity, based on the clinical evaluation performed by the attending psychiatrist and further supported by the scores obtained on the administered psychiatric rating scale. None of these patients presented psychotic features, and therefore, antipsychotic treatment was not clinically indicated. Therapeutic management was established by the attending psychiatrist according to an individualized treatment approach; in general, treatment regimens consisted of either an SSRI combined with an anxiolytic agent or a dual-action antidepressant. The SSRIs used included: Sertraline (starting with 50 mg and raising to 200 mg), Escitalopram (starting with 10 mg and raising to 20 mg), Fluvoxamine (starting with 50 mg and raising to 200 mg), and Paroxetine (starting with 20 mg and raising to 60 mg). The anxiolytics used included: Lorazepam (starting with 1 mg and raising to 4 mg), Clonazepam (starting with 0.5 mg and raising to 4 mg), and Bromazepam (starting with 1.5 mg and raising to 6 mg). The dual-action antidepressants included: Mirtazapine (starting with 30 mg and raising to 45 mg), Venlafaxine (starting with 75 mg and raising to 300 mg), Doxepin (starting with 25 mg and raising to 100 mg), Clomipramine (starting with 25 mg and raising to 100 mg), Trazodone (starting with 50 mg and raising to 450 mg), and Bupropion (starting with 150 mg and raising to 450 mg).

Patients diagnosed with GAD were also undergoing their first psychiatric hospitalization and symptom severity was determined by clinical assessment and confirmed using the corresponding psychiatric rating scale. In this group, treatment was individualized by the attending psychiatrist and generally consisted of anxiolytic medication alone.

All patients diagnosed with BD were admitted during a manic episode, clinically established by the attending psychiatrist and supported by the administered clinical rating scale. None of the BD patients presented psychotic features; consequently, antipsychotic medication was not initiated. For mood stabilization and prevention of affective switching, the attending psychiatrist prescribed mood stabilizer monotherapy, consisting of either lithium (starting with 400 mg and raising to 1600 mg) or valproate (starting with 500 mg and raising to 2000 mg). In patients with BD, lithium treatment was initiated after baseline thyroid blood sampling, thereby minimizing the likelihood that lithium exposure during the current admission influenced the thyroid parameters analyzed in this study.

### 2.3. Eligibility Criteria

The inclusion criteria were patients aged over 18 years old and above 65 years; primary diagnosis of MDD, GAD, or BD; hospitalized for acute psychiatric symptoms; consent and capacity to provide informed consent (or consent of legal guardian, if applicable).

Patients older than 65 years were not included in order to reduce the potential confounding effect of age-related cognitive impairment. In this age group, affective and anxiety symptoms may overlap with mild cognitive impairment or early neurocognitive disorders, which could have influenced psychiatric scale scores and diagnostic classification. Patients with previously documented primary thyroid disorders, including hypothyroidism, hyperthyroidism, or autoimmune thyroiditis, were excluded from the study. Patients with severe acute medical conditions were also excluded because these conditions may alter thyroid test values in the absence of actual endocrine pathology. In terms of other comorbidities, the patients had controlled type 2 diabetes mellitus or stabilized hypertension. Pregnancy is another exclusion criterion due to specific hormonal features. In addition, individuals taking medications known to interfere with thyroid function tests of thyroid hormone metabolism, such as amiodarone, lithium, systemic glucocorticoids, dopamine agonists, iodine-containing preparations, interferon-alpha, interleukin-2, and antiepileptics were excluded.

### 2.4. Thyroid Function Assessment

Serum levels of thyroid-stimulating hormone (TSH), free thyroxine (FT4), and free triiodothyronine (FT3) were obtained 24–48 h after admission to minimize NTI influence. Also, the blood samples for thyroid assessment were obtained before the initiation of lithium or other mood stabilizer treatment. Although total T3 and T4 can also be assayed, free FT4 and FT3 are preferred because they represent the biologically active, unbound fractions of thyroid hormones [9]. Measurements were performed using standardized immunoassay methods, performed in a single accredited laboratory, to ensure consistency of results and limit intra-assay variation. However, it should be noted that thyroid function tests are not completely standardized globally, and differences in methodology and type of antibodies used may result in slight variations between techniques [10].

Based on serum TSH, FT4, and FT3 levels, using the laboratory reference range (with a normal TSH value between 0.4 and 4.5 mIU/L), participants were classified according to thyroid functional status as follows: euthyroidism, overt hypothyroidism, subclinical hypothyroidism, overt hyperthyroidism, and subclinical hyperthyroidism.

### 2.5. Clinical and Psychiatric Assessment

To quantify the severity of psychiatric symptoms, standardized, validated and internationally recognized instruments were used:

- for MDD: the Montgomery-Åsberg Depression Rating Scale (MADRS)—that is a 10-item clinical instrument with a total score ranging from 0 to 60 and is designed to assess the core emotional symptoms of depression. This scale is frequently used in clinical research due to its sensitivity to changes in symptomatology and is suitable for objective assessments in the hospital setting [11];
- for GAD: the Hamilton Anxiety Rating Scale (HAM-A)—includes 14 items, with a total score ranging from 0 to 56 and is a widely used instrument in clinical practice to assess the severity of anxiety symptoms, based on clinician observation [11]. Due to its comprehensive nature, the HAM-A is considered a standard for the clinical assessment of anxiety [12];
- for BD: the Young Mania Rating Scale (YMRS)—composed of 11 items and administered by the clinician—is recognized as the gold standard in assessing the severity of manic symptoms [6].

### 2.6. Study Variables and Data Collection

Demographic information on participants (including age, gender, primary psychiatric diagnosis, duration of current illness episode, and current psychotropic treatment) was obtained through a mixed methodology combining structured interviews with patients and electronic medical records. Thyroid hormone levels were obtained from the designated clinical laboratory, following blood sampling. Psychometric instruments were administered by trained and qualified medical personnel (for clinical scales).

### 2.7. Statistical Analysis

Descriptive statistics:

- For continuous variables (such as hormone levels and scores obtained on psychometric scales), the mean, standard deviation (SD), median and interquartile range (IQR) were reported.
- Categorical variables (such as demographic data or distribution by diagnostic group) were described by absolute frequencies and percentages. Assessment of the normal distribution of the data was performed by the Shapiro–Wilk test and analysis of skewness and kurtosis coefficients.

Comparative analyses:

- Between diagnostic groups: One-way analysis of variance (ANOVA) was applied to compare mean thyroid hormone values and psychometric scores between the three main diagnostic categories (MDD, GAD, BD). For variables that did not meet the normality assumption, the Kruskal–Wallis test was used as the appropriate non-parametric alternative. If the overall analysis indicated significant differences, appropriate post hoc tests were performed, such as Tukey’s HSD (for ANOVA) or the Dunn test (for Kruskal–Wallis);
- Between thyroid function categories: The chi-square test was used to compare the distribution of primary psychiatric diagnoses across thyroid functional status groups.

Correlation analysis: Associations between continuous thyroid hormone levels (TSH, FT4, and FT3) and psychiatric scale scores (MADRS, HAM-A, and YMRS) were assessed using Pearson’s correlation coefficient for normally distributed variables and Spearman’s rank correlation coefficient when the normality assumption was not met.

Regression analysis: Multiple linear regression models were used to evaluate whether thyroid function independently contributed to psychiatric symptom severity. Separate models were initially conducted according to diagnostic group, using the corresponding clinical severity scale as the dependent variable: HAM-A for generalized anxiety disorder, MADRS for major depressive disorder, and YMRS for bipolar disorder. Thyroid-related variables, including serum TSH, FT3, FT4, and thyroid status, were included as predictors, together with relevant covariates such as age, sex, episode duration, and current medication type.

## 3. Results

### 3.1. Descriptive Statistics

The study included a group of 88 patients admitted to a psychiatric ward. Within this sample, the youngest patient was 18 years old and the oldest 65 years old, with a mean age of 42.5 years and a standard deviation of ±12.3 years, suggesting a relatively wide distribution within the group. Depending on age categories, 22.7% had between 18 and 29 years old, 34.10% had between 30 and 44 years old, 29.5% had between 45 and 59 years old, and 13.60% had between 60 and 65 years old.

Of the total of 88 patients included, 56 (63.6%) were women and 32 (36.4%) were men.

The duration of the current psychopathological episode was on average 6.8 months (with a standard deviation of 3.5 months and an estimated range of 3.3 to 10.3).

In terms of categorising the group based on the primary diagnosis, the number of patients with MDD was 30 (34.1%), the number of patients with GAD was 30 (34.1%), and the number of patients with BP was 28 (31.8%).

Regarding pharmacological treatment, we can see the distribution of patients based on the therapeutic regimens. So, out of the 30 patients with MDD, 21 (70%) needed a dual-action antidepressant, while 9 (30%) needed a combination of SSRI and anxiolytic. All the patients with GAD received only anxiolytics. Out of the 28 patients with BD, 20 patients received Lithium as a mood stabilizer and 8 received Valproate (Table 1).

The distribution of patients across severity categories based on the applied clinical rating scales are:

- MADRS: Based on MADRS scores, moderate depressive symptoms predominated across the diagnostic groups. In the MDD group, all patients showed at least moderate depressive symptomatology, with 18 cases classified as moderate and 12 as severe; no cases of mild depression were identified in this group. Among patients with GAD, 20 presented moderate depressive symptoms and 10 had mild depressive symptoms, with no cases classified as severe. In the BD group, 26 patients presented moderate depressive symptoms, and 2 had mild depressive symptoms.
- HAM-A: Based on HAM-A scores, patients with GAD predominantly presented severe or very severe anxiety, with 14 cases classified as very severe and 12 as severe; only 4 patients showed moderate anxiety. Among patients with MDD, anxiety severity was distributed mainly across moderate and severe levels, with 12 and 10 cases, respectively, while 6 patients had mild anxiety and 2 presented very severe anxiety. In the BD group, anxiety symptoms were most frequently of moderate severity, observed in 14 cases, followed by severe anxiety in 8 cases and mild anxiety in 4 cases; 2 patients presented very severe anxiety.
- YMRS: Among patients with BD, YMRS scores indicated that manic symptoms were most frequently classified as severe or moderate, with 12 and 10 cases, respectively. Mild manic symptoms were recorded in 4 patients, while 2 patients had no clinically significant manic symptoms.

Table 2 presents the means and standard deviations for thyroid hormone levels and psychiatric scale scores, both for the entire cohort and broken down by primary diagnostic group.

**Figure 2:**
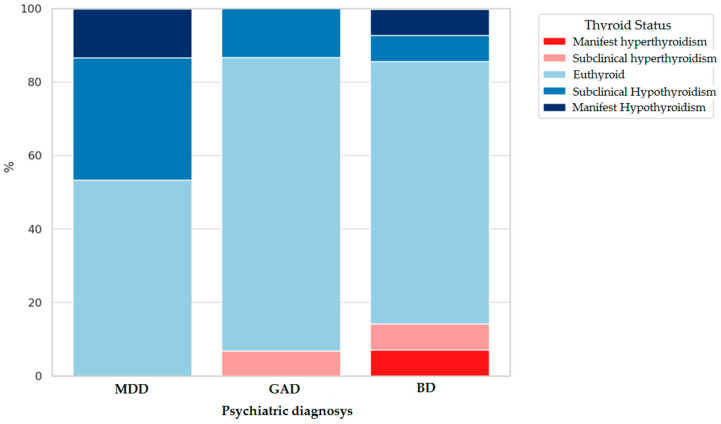
Distribution of thyroid status by diagnostic groups.

Table 3 and Figure 2 present the prevalence of each thyroid status category in the overall cohort, stratified by primary psychiatric diagnosis.

### 3.2. Comparative Analysis

ANOVA analysis revealed statistically significant differences in mean TSH values between diagnostic categories (*p* < 0.01), with patients with MDD presenting significantly higher mean TSH levels compared to those diagnosed with GAD and BD, according to the results of post hoc tests (*p* < 0.05). In contrast, for FT4 and FT3, no statistically significant variations were identified between groups. Regarding psychiatric severity scores, significant differences were noted between groups for MADRS (*p* < 0.001) and HAM-A (*p* < 0.001), indicating distinct symptomatic profiles associated with each diagnosis.

**Figure 3:**
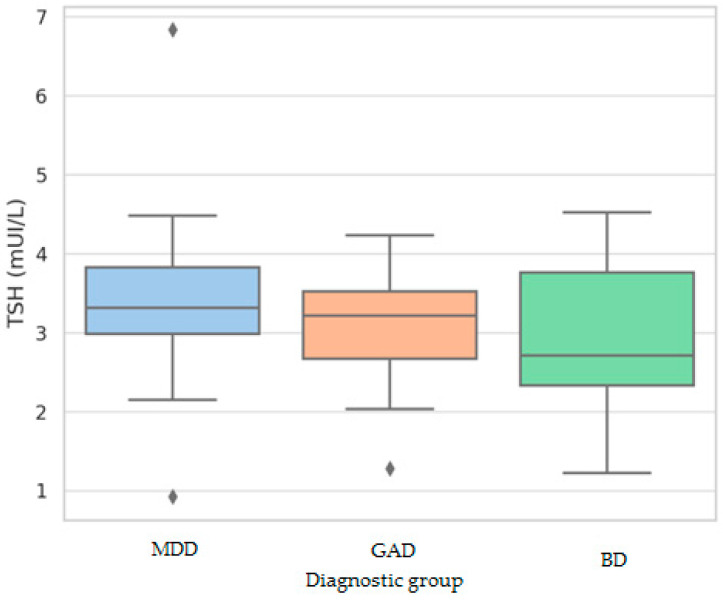
Distribution of TSH values.

**Figure 4:**
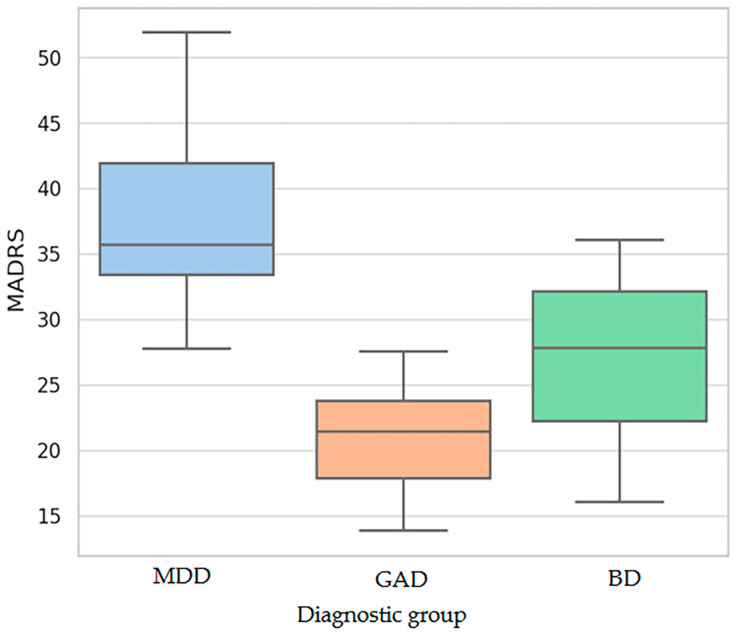
Distribution of MADRS values.

**Figure 5:**
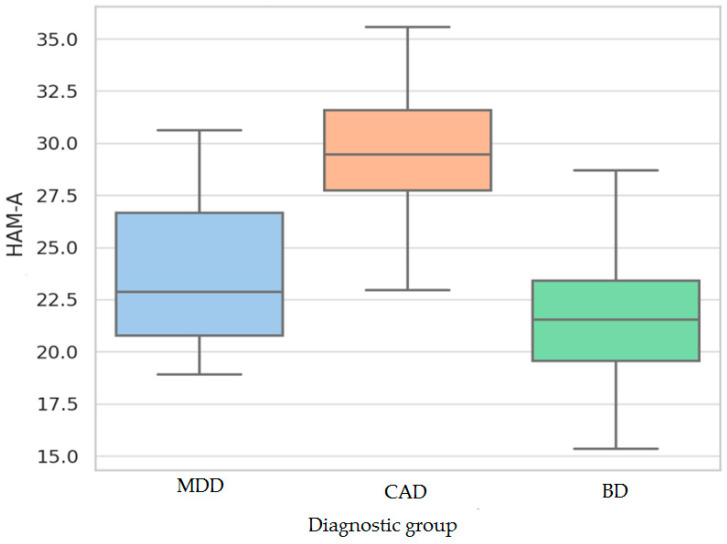
Distribution of HAM-A values.

To complete the numerical interpretation presented in Table 2, box plots were constructed for the TSH (Figure 3), MADRS (Figure 4) and HAM-A variables (Figure 5), depending on the diagnostic group. These graphs provide a visual validation of the statistically significant results obtained by ANOVA.

The MDD group presented higher median values of TSH and significantly greater variability, suggesting the presence of outliers or a broader distribution.

The MDD group had clearly higher median scores of MADRS, which visually validates the significant ANOVA differences.

The group with GAD had the highest median scores of HAM-A, with relatively low dispersion, highlighting marked and consistent anxiety.

Chi-square test indicated a statistically significant association between primary psychiatric diagnosis and thyroid status (*p* < 0.05). In particular, patients with MDD showed a significantly higher prevalence of hypothyroidism (combined subclinical and overt) compared to patients with GAD and BD.

To address the potential influence of mood stabilizer treatment on thyroid parameters in the BD subgroup, thyroid indices were compared between patients receiving lithium and those receiving valproate. Independent-samples comparisons were performed for TSH, FT4, and FT3 (Table 4).

No statistically significant differences were observed between the lithium and valproate groups for TSH or FT3 levels. FT4 levels were significantly higher in the lithium group compared with the valproate group. Therefore, lithium treatment did not appear to account for the observed differences in TSH or FT3 within the BD subgroup, although the difference in FT4 should be interpreted cautiously given the small number of valproate-treated patients and the observational design of the study.

### 3.3. Correlation Analysis

Table 5 presents the Pearson correlation matrix between thyroid hormone levels and psychiatric scale scores.

The correlation results indicate a significant positive correlation between TSH and MADRS scores (r = 0.45, *p* < 0.05) and HAM-A (r = 0.38, *p* < 0.05), suggesting that higher TSH levels are associated with greater severity of depressive and anxiety symptoms.

In contrast, FT4 and FT3 showed significant positive correlations with YMRS scores (FT4: r = 0.30, *p* < 0.05; FT3: r = 0.42, *p* < 0.05), indicating that higher levels of these free hormones are associated with increased severity of manic symptoms.

**Figure 6:**
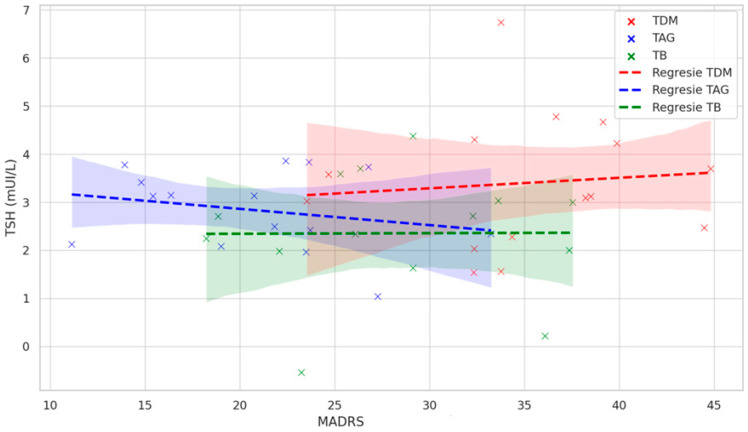
Relationship between TSH level and MADRS score, segregated by diagnosis (MDD, GAD, BD).

If we look at the relationship between TSH level and MADRS score, segregated by diagnosis (MDD, GAD, BD), a notable positive correlation between MADRS and TSH is observed among patients with MDD, while this relationship is much weaker or non-existent in the GAD and BD groups (Figure 6). This suggests that thyroid changes may have a differentiated clinical relevance depending on the underlying pathology.

**Figure 7:**
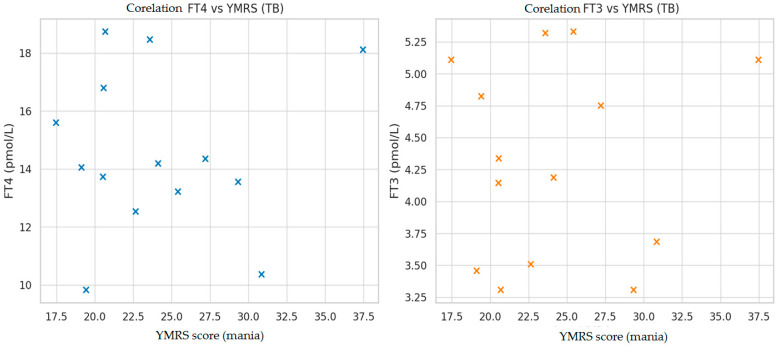
Correlation between FT4/FT3 and YMRS score in bipolar disorder (N = 28).

There is a general positive trend between elevated FT4 and FT3 values and higher YMRS scores, suggesting a possible influence of thyroid hyperactivity on the intensity of manic symptoms (Figure 7).

### 3.4. Regression Analysis

One of the main objectives of our study was to examine whether we can predict the severity of the affective disorder based on personal factors, disease factors and thyroid status. To this end, we conducted several categorical regression analyses, which had as a dependent variable the clinical score at the severity scales for each of the diagnoses (MADRS for MDD, HAM-A for GAD, and YMRS for BD). The independent variables introduced in the analyses were: age, sex, episode duration, medication type, TSH, FT3, FT4, and thyroid status as a categorial variable. However, none of these diagnosis-specific models reached statistical significance. The MDD model showed only a trend toward significance (R^2^ = 0.556, adjusted R^2^ = 0.309, *p* = 0.064). The GAD model was not significant (R^2^ = 0.294, adjusted R^2^ = −0.023, *p* = 0.524), and neither did the BD model (R^2^ = 0.281, adjusted R^2^ = −0.213, *p* = 0.827). Therefore, these initial full models were considered insufficiently robust, likely due to the relatively small sample size within each diagnostic subgroup and the high number of predictors included.

Subsequently, because the MDD subgroup showed the strongest signal in the initial exploratory analysis, additional regression models were tested in this subgroup. In the first MDD-specific model, thyroid status was entered using the original five-category classification: euthyroidism, subclinical hypothyroidism, overt hypothyroidism, subclinical hyperthyroidism, and overt hyperthyroidism. However, this model did not reach statistical significance at the conventional threshold, although it showed a trend toward significance (R^2^ = 0.556, adjusted R^2^ = 0.309, *p* = 0.064). Given the limited number of patients in some thyroid dysfunction categories, thyroid status was recoded into three clinically meaningful categories: euthyroidism, hypothyroidism, and hyperthyroidism. Hypothyroidism included both subclinical and overt hypothyroidism, while hyperthyroidism included both subclinical and overt hyperthyroidism. This recoding was performed to reduce category fragmentation and improve model stability.

In patients with MDD, a multiple linear regression analysis was performed using the MADRS score as the dependent variable.

The initial model included age, sex, episode duration, medication type, and thyroid status. Medication type was recorded into two categories: patients with dual-action antidepressant medication and patients with a combination of SSRI and anxiolytic treatment. Thyroid status was recorded into three categories: patients with euthyroidism, patients with hypothyroidism, and patients with hyperthyroidism. This initial model was statistically significant and explained approximately 51.2% of the variance in MADRS scores (Table 6).

The overall model was statistically significant: F(6,22) = 3.844, *p* = 0.009 (Table 7).

Table 8 shows the regression coefficients together with the *t*-tests, which helped us develop the multiple regression equation for the dependent variable. In the initial model, medication type was not a significant predictor of MADRS score, B = 1.704, *p* = 0.537. The significant predictors were male sex and hypothyroidism (Table 8).

The final regression model used backward elimination and removed the non-significant variables, including age, episode duration, and medication type. The final model retained sex and thyroid status recoded into three categories. This final model explained 48.5% of the variance in the MADRS scores, with an adjusted R^2^ of 0.423 (Table 9).

The final model was statistically significant: F(3,25) = 7.853, *p* = 0.0007 (Table 10).

Table 11 shows the regression coefficients together with the *t*-tests, which helped us develop the multiple regression equation for the dependent variable (Table 10).

The final model obtained through backward elimination retained sex and thyroid status and remained statistically significant, F(3,25) = 7.853, *p* = 0.0007, R^2^ = 0.485, adjusted R^2^ = 0.423. Hypothyroidism was significantly associated with higher MADRS scores compared with euthyroidism, B = 6.036, 95% CI [1.212, 10.859], *p* = 0.016. Male sex was also associated with higher MADRS scores, B = 6.343, 95% CI [1.708, 10.977], *p* = 0.009. Hyperthyroidism showed a positive but non-significant association with MADRS scores, B = 6.755, 95% CI [−1.687, 15.196], *p* = 0.112.

Because the initial diagnosis-specific regression model was not statistically significant and only the simplified MDD model reached significance, these regression results should be considered exploratory and hypothesis-generating rather than confirmatory.

## 4. Discussion

Our study evaluated 88 inpatients with three affective disorders (MDD, GAD, and BD) within a single assessment, linking TSH, FT4, and FT3 with standardized clinician scales (MADRS, HAM-A, and YMRS). Our objectives were to characterize the thyroid hormonal profiles of these patients and to characterize the clinical severity of their symptoms. Our main objective was to evaluate whether thyroid dysfunction predicted disorder-specific clinical severity scores after accounting for age, sex, episode duration, medication type, and thyroid hormone profiles.

The first important result was that the mean TSH is significantly higher in MDD compared with GAD and BD. On the other hand, no statistically significant differences were found among the diagnostic subgroups based on the values of FT3 and FT4.

The second important result was the positive correlation between TSH and the depression scale (MADRS) and the anxiety scale (HAM-A). So, higher TSH levels are associated with greater severity of depressive and anxiety symptoms. On the other hand, there was a positive correlation between FT3 and FT4 with the mania scale (YMRS). So, higher free hormone levels are associated with higher mania.

Our results highlight the existence of a complex and multifactorial relationship between thyroid function parameters and the severity of affective symptomatology in patients admitted to a psychiatric unit. Although the majority of patients presented with euthyroid thyroid function, a significant percentage showed thyroid dysfunction, with an increased incidence of subclinical hypothyroidism among those diagnosed with major depressive disorder. The positive association between TSH values and depression severity scores (MADRS) and anxiety (HAM-A) is consistent with the literature, which indicates that elevated TSH levels are often correlated with hypothyroidism and negative affective symptomatology, such as apathy, cognitive impairment, and depressive mood [13]. Overt hypothyroidism is characterized by a variety of symptoms including cognitive impairment and dysphoric states, and in the case of subclinical forms, the risk of developing depression is up to four times higher, especially in the elderly population [14].

A direct correlation was observed between FT4 and FT3 values and scores related to manic symptomatology, clinically evaluated with YMRS, which supports the hypothesis that thyroid hyperactivity could favour the appearance of psychomotor agitation, pathological euphoria and even psychotic disorders [14]. Moreover, a study published in 2021 revealed significant variations in thyroid hormone levels depending on the phase of the affective episode in patients with bipolar disorder, with increased thyroid hormone levels in manic phases compared to depressive ones [15].

The increased prevalence of hypothyroidism, both in its manifest and subclinical form, in the group of patients with MDD supports a strong and well-documented association between this endocrine condition and depression [14]. Previous studies have repeatedly described a significant comorbidity between thyroid dysfunctions and affective disorders, suggesting a possible common etiopathogenic mechanism [16].

Mood disorders are more common in women than in men, this difference being attributed to both biological and hormonal factors, as well as psychosocial ones. In our study, this trend is confirmed with a total of 88 patients included, 56 (63.6%) are women, and 32 (36.4%) are men. This predominance of the female sex can be explained by a number of factors, including a higher probability of women seeking medical help for affective symptoms. However, although this distribution is consistent with general epidemiological trends, the findings of our study should not be generalized to the entire population without considering the specific characteristics of the study sample. On the other hand, our study suggested that male sex was independently associated with higher MADRS scores in the final regression model. Although depressive disorders are generally reported more frequently among women, this finding may reflect sex-related differences in help-seeking behavior rather than a higher overall burden of depression in men. Previous studies have shown that men are less likely to seek professional help for depressive symptoms and may delay access to mental health care, which could result in more severe clinical presentations by the time they are evaluated in psychiatric settings [17]. Therefore, the association between male sex and higher depressive severity in the present sample may indicate a selection effect, whereby men included in clinical samples are more likely to present with more severe symptomatology. However, the regression analyses should be interpreted as exploratory. Although the initial diagnosis-specific models did not reach statistical significance, the simplified MDD model suggested that hypothyroid status and male sex may be associated with greater depressive symptom severity. This finding may indicate that the relationship between thyroid dysfunction and affective symptom burden is more detectable within depressive presentations than across broader diagnostic categories. However, the fact that significance emerged only after model simplification suggests that these results should be viewed as hypothesis-generating rather than confirmatory.

The literature also increasingly foregrounds bidirectional and temporal associations: for instance, a recent large prospective study, with approximately 350,000 participants, found that higher depression and anxiety scores predicted future development of hypothyroidism and hyperthyroidism over 13 years [18]. That raises the possibility that mood and anxiety disturbances may drive thyroid perturbations in some individuals, not only the reverse. Autoimmune thyroiditis and TPO/Tg-antibody positivity have been investigated as moderators of mood–thyroid associations, though population meta-analyses are equivocal about their independent effect on depression [19]. The well-established concept of non-thyroidal illness remains an important methodological consideration in endocrine–psychiatric research, as systemic illness can alter thyroid laboratory parameters, particularly T3 levels, thyroid hormone binding, and free hormone fractions [20].

In the last 10 years, the relationship between thyroid function and mood or anxiety disorders has remained an active area of research, yet one still marked by heterogeneity, nuance, and sometimes contradictory findings. Many research studies frame the thyroid–psychiatric link in several situations: subclinical hypothyroidism associated with depression, phase-dependent thyroid shifts in bipolar disorder, and the ever-present spectre of non-thyroidal illness complicating interpretation. For example, a meta-analysis pooling over 100,000 individuals concluded that subclinical hypothyroidism is positively associated with depression in adults, especially those over age 50 [21]. Other large-scale cross-sectional analyses, including NHANES-based studies, have similarly shown modest but significant correlations between depression symptom scores and thyroid hormone levels (FT3, FT4) that sometimes differ by age or sex strata [22]. Yet, when meta-analyses probe the link between clinical depression and overt or subclinical hypothyroidism in population cohorts, the effect sizes shrink; one large systematic review found a moderate association for overt hypothyroidism, but only a borderline link for subclinical forms, with stronger signals in female subgroups [23].

In bipolar disorder, recent meta-analyses and comparative studies have documented shifts in thyroid hormones by mood state: one 2024 meta-analysis showed that in manic states, FT4 tends to increase while T3 declines (versus healthy controls), and T3/FT3 are lower in bipolar depression relative to mania [24]. Other scientific articles suggest that thyroid status anomalies—especially in free hormone levels or deiodinase activity—may differ across phases, and these differences often fluctuate with medication status and chronic illness [25]. Other studies highlighted that elevated systemic inflammatory indices, including CRP, Neutrophil-to-Lymphocyte Ratio, and Systemic Immune-Inflammation Index, may act as systemic modulators of psychiatric phenotypes, a perspective that complements our proposition that thyroid perturbations in affective disorders may partly reflect immune–endocrine cross-talk rather than isolated gland dysfunction [26].

Beyond the established influence of thyroid dysfunction on mood and anxiety symptoms, growing evidence suggests that psychiatric disorders themselves may contribute to alterations in thyroid function through neuroendocrine stress responses, inflammatory mechanisms, and immune-mediated pathways. Abnormal thyroid hormone profiles appear to be relatively common among patients with severe psychiatric disorders, including schizophrenia-spectrum disorders and mood disorders. Thyroid dysfunction has been reported in approximately 29% of patients with schizophrenia-spectrum disorders and 23% of those with mood disorders, while autoimmune thyroid disease appears to be more frequent among patients with schizophrenia-spectrum disorders [27]. There is emerging evidence of shared genetic susceptibility, immune-mediated pathways and altered hypothalamic–pituitary–thyroid (HPT) axis regulation. A recent study found significant genetic correlations between hypothyroidism and psychiatric disorders such as MDD, anxiety disorders and schizophrenia, indicating common pleiotropic risk loci [28]. Moreover, in depressed patients, alterations such as blunted TSH response to TRH, absence of nocturnal TSH surge, and subtle shifts in free T4/TSH have been documented, suggesting secondary HPT-axis dysregulation [29].

**Figure 8:**
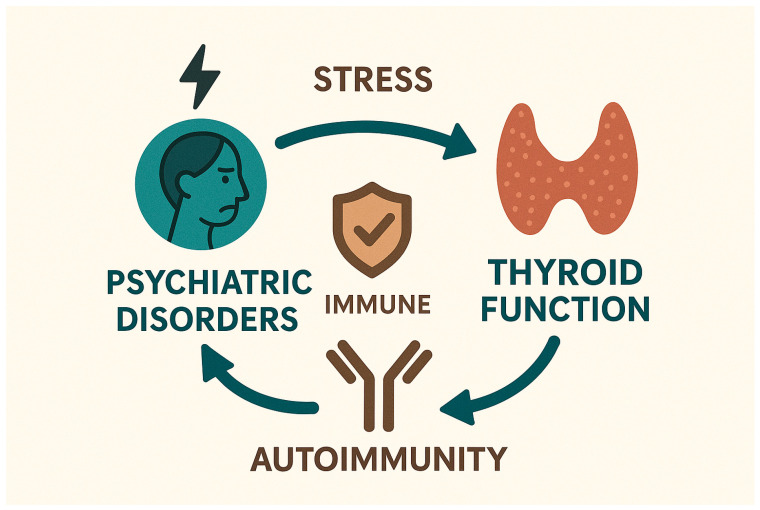
Bidirectional interaction between psychiatric disorders and thyroid function.

Immune–thyroid crosstalk provides another route by which psychiatric disease can impair thyroid function. Pro-inflammatory cytokines (e.g., IL-6, TNF-α), which are often elevated in mood disorders, shift peripheral deiodinase activity, producing a low-T3, non-thyroidal illness pattern and reduced tissue thyroid signalling without primary gland failure [30]. In parallel, mood and anxiety disorders show links with thyroid autoimmunity: population and clinical studies report higher odds of anti-TPO/anti-Tg antibodies in depressed/bipolar cohorts, including youth, suggesting that autoimmune activity may be part of the shared pathobiology [31]. Together, stress hormones, cytokine-driven deiodinase changes, and autoimmunity explain how psychiatric disorders can secondarily suppress thyroid function. Conversely, primary thyroid disease (hypo- or hyperthyroidism) can precipitate or worsen depressive, anxious, cognitive, or manic symptoms—completing a biologically bidirectional loop [30,32,33,34,35] (Figure 8).

From a clinical point of view, this means that the relationship is bidirectional: while overt thyroid disease can lead to psychiatric symptoms (hypothyroidism can lead to depression or hyperthyroidism can lead to anxiety and mania), also psychiatric conditions themselves may impair thyroid regulation, perhaps via stress, immune activation or medication effects [36]. The implication is that in patients with major psychiatric illness, especially if treatment-resistant or with atypical features, assessing thyroid status (including subtle HPT axis markers) is needed. Likewise, thyroid abnormalities in a psychiatric patient should not be dismissed, as they may contribute to the illness presentation and course.

Also, across bipolar, depressive, and anxiety disorders, patients frequently show shared HPA-axis dysregulation: higher or dysregulated cortisol, loss of normal circadian dynamics, and blunted feedback. This stress biology alters monoamine signalling and neuroplasticity in overlapping ways, helping explain why affective and anxiety symptoms often co-occur within the same person. Systematic reviews document that HPA abnormalities in MDD and BD are strongly shaped by stress exposure and trauma, supporting a transdiagnostic mechanism [37]. A second transdiagnostic layer is immune activation. Meta-analyses and cohort studies consistently find elevated IL-6 and TNF-α in MDD, with similar inflammatory signals reported in BD; these cytokines can drive sickness behavior, anhedonia, anxiety, and cognitive changes that blur categorical boundaries. Immune signalling also interfaces with endocrine axes (including the thyroid), producing “low-T3–like” states and other subtle HPT-axis shifts sometimes seen in mood and anxiety disorders—again yielding overlapping clinical pictures [38,39,40,41]. Genetic studies show that these disorders partly share risk architecture (pleiotropic loci and correlated polygenic signals), implying common upstream biology that can manifest as mixed affective–anxiety phenotypes in the same individual. This shared genetic liability likely channels through stress-response and immune pathways described above, reinforcing comorbidity [42,43].

An additional consideration is the potential overlap between diagnostic categories within the studied cohort. Although patients were classified according to a primary psychiatric diagnosis, affective disorders frequently present with comorbid or overlapping symptomatology, particularly between MDD, GAD, and BD. Anxiety symptoms are highly prevalent in depressive episodes, while depressive features are common in both bipolar and anxiety disorders, which may blur diagnostic boundaries in a cross-sectional clinical setting [44,45]. This dimensional overlap may partly explain the shared associations observed between thyroid parameters and symptom severity across diagnostic groups, suggesting that endocrine alterations could be linked more closely to transdiagnostic symptom domains—such as emotional distress or illness severity—rather than to categorical diagnoses alone. These considerations highlight the importance of adopting a dimensional and integrative approach when interpreting the relationship between thyroid function and psychiatric disorders, particularly in heterogeneous inpatient populations.

On the other hand, variations in thyroid function, even in the absence of overt pathology, may have significant implications for mental health. Hormonal changes at the lower or upper limits of reference ranges have been associated with an increased risk for the development of common psychiatric disorders, including depression and anxiety [46]. Thus, a nuanced clinical approach is required, which includes dynamic and contextualized assessment of thyroid function, in order to identify patients who could benefit from specific interventions or integrated endocrinological monitoring. However, a recent systematic review and meta-analysis reported significantly lower TSH concentrations in drug-naïve patients with first-episode psychosis compared with healthy controls, supporting the hypothesis that early severe psychiatric illness may involve alterations of anterior pituitary hormone secretion and hypothalamic–pituitary–thyroid axis regulation [47]. In this context, the association between MDD and thyroid dysfunction may be particularly relevant, as depressive severity is not limited to affective symptoms but is also closely linked to fatigue, apathy, and cognitive impairment, suggesting that thyroid alterations could contribute to a broader clinical phenotype characterized by both emotional and cognitive burden [48].

The observed associations between thyroid hormone parameters and affective symptom severity also raise important considerations regarding the therapeutic role of thyroid hormones in psychiatric disorders. Thyroid hormone supplementation, particularly with levothyroxine (T4) or liothyronine (T3), has been investigated as an adjunctive treatment in mood disorders, especially in treatment-resistant depression and certain phases of bipolar disorder [49]. Evidence suggests that augmentation with T3 may accelerate antidepressant response and enhance treatment efficacy, even in patients with baseline euthyroid function [50]. In bipolar disorder, supraphysiological doses of thyroid hormones have been explored in refractory cases, with some studies indicating a potential benefit in stabilizing mood and reducing rapid cycling [51]. However, these interventions remain controversial due to variability in clinical response and the risk of adverse effects, including iatrogenic hyperthyroidism and cardiovascular complications. In this context, the present findings support a more individualized and biologically informed approach, where thyroid function assessment may help identify subgroups of patients who could benefit from targeted endocrine interventions, while emphasizing the need for careful monitoring and further longitudinal studies to clarify efficacy and safety.

Several limitations of our study should be noted. First, the observational, cross-sectional design provides a view at a given point in time and therefore cannot establish causal relationships or temporal sequences between exposure (thyroid hormone levels) and outcome (severity of psychiatric symptoms). Second, the small size of the participant group considerably restricts the statistical power of the analyses, reducing the ability to identify subtle relationships or significant differences between clinical subgroups. This limitation also negatively affects the possibility of extrapolating the results to larger populations or to the outpatient setting, where the symptomatic profile and medical context may differ significantly [5]. On the other hand, the exclusive focus on the thyroid axis, without including other relevant hormonal markers in the analysis, such as cortisol (HPA axis) or gonadal hormones (HPG axis), is also a limitation. Future research should aim for a multi-hormonal approach to provide a more integrative view of the endocrine dysfunctions in affective disorders. In addition, the determination of anti-thyroid peroxidase antibodies (TPOAb) and anti-TSH receptor antibodies (TRAb/TSI) would provide additional information, providing relevant clues about the presence of autoimmune thyroiditis, a condition frequently associated with affective disorders, sometimes even in the absence of obvious hormonal changes [1]. However, because TPOAb were not measured in the present study, previously undiagnosed autoimmune thyroiditis could not be excluded, even though patients with a known history of autoimmune thyroid disease were not included. Autoimmune thyroiditis may be present even in euthyroid patients, but it may also underlie thyroid dysfunction. Therefore, the absence of antibody assessment limits the ability to distinguish non-autoimmune thyroid dysfunction from autoimmune thyroid disease, and some thyroid abnormalities observed in this cohort may have reflected unrecognized autoimmune thyroid dysfunction. While mentioning the instruments that we used, there may be other alternative instruments that would have provided a better understanding of our group such as: the Patient Health Questionnaire-9 (PHQ-9) and the Quick Inventory of Depressive Symptomatology Self-Report (QIDS-SR16) for depression [11]; the Generalized Anxiety Disorder 7-item Scale (GAD-7), for rapid screening of anxiety [12]; and the Mood Disorder Questionnaire (MDQ), for specific symptomatology of BD [1]. Another important limitation is related to the possibility that thyroid alterations represent non-thyroidal illness (NTI), in which thyroid changes, such as decreased T3 levels, represent an adaptive response to the physiological stress of acute psychiatric illness rather than a primary thyroid dysfunction. The severity of these changes is often proportional to the intensity of psychopathology, suggesting that abnormal thyroid values may be secondary indicators of the severity of the underlying illness, rather than direct causal factors [52]. For example, an association between low T3 levels and high depression scores could reflect the general severity of psychiatric illness, without necessarily implying a direct causal relationship between thyroid parameters and affective symptomatology [6,53]. In addition, although thyroid blood samples were collected early during hospitalization, within 24–48 h after admission, and before the initiation of mood stabilizer treatment in patients with BD, some patients may have already received initial doses of antidepressant or anxiolytic medication before blood sampling. Therefore, while this sampling window was chosen to limit acute NTI-related fluctuations and to obtain an early endocrine profile, a potential early medication-related effect on thyroid hormone parameters cannot be completely excluded. This should be considered when interpreting the endocrine findings.

## 5. Conclusions

The present study supports a clinically relevant association between thyroid function and affective symptom severity in psychiatric inpatients. Higher TSH levels were particularly linked to depressive and anxiety symptom burden, while higher FT3 and FT4 levels were associated with manic symptom severity, suggesting that thyroid alterations may relate differently to distinct affective dimensions. Although most patients were euthyroid, thyroid dysfunction was relatively frequent, especially hypothyroid patterns among patients with MDD. Regression analyses further suggested that thyroid status, particularly hypothyroidism, may independently contribute to depressive severity after accounting for relevant clinical variables. These findings reinforce the importance of routine thyroid assessment in patients with mood and anxiety disorders and support a more integrated endocrine–psychiatric approach, as thyroid dysfunction may contribute to both the pathogenesis and clinical severity of these conditions.

## Figures and Tables

**Table 1:** Pharmacologic treatment distribution.

Type of Medication	No. of Patients	Diagnostic
Dual-action antidepressant	21 out of 30 (70%)	MDD
SSRI + anxiolytic	9 out of 30 (30%)	
Anxiolytics	30	GAD
Mood stabiliser (Lithium)	20 out of 28 (71.42%)	BD
Mood stabiliser (Valproate)	8 out of 28 (28.57%)	

**Table 2:** Thyroid hormone levels and psychiatric scale scores.

Variable	Total (*n* = 88)	MDD (*n* = 30)	GAD (*n* = 30)	BD (*n* = 28)
TSH (mUI/L)	3.2 ± 1.5	4.1 ± 1.8	2.8 ± 1.0	2.6 ± 1.2
FT4 (pmol/L)	14.5 ± 2.1	13.8 ± 1.9	15.1 ± 2.0	14.9 ± 2.2
FT3 (pmol/L)	4.2 ± 0.8	3.9 ± 0.7	4.4 ± 0.8	4.3 ± 0.9
MADRS (score)	28.1 ± 7.5	35.2 ± 6.1	22.5 ± 5.8	26.6 ± 7.0
HAM-A (score)	25.3 ± 6.2	23.8 ± 5.9	30.1 ± 4.5	21.5 ± 5.5
YMRS (score)	12.8 ± 8.0	N/A *	N/A *	25.5 ± 7.2

N/A *: Not applicable for the respective diagnoses.

**Table 3:** Distribution of thyroid status according to primary psychiatric diagnosis.

Thyroid Status (*n*, %)	Total Cohort (*n* = 88)	MDD (*n* = 30)	GAD (*n* = 30)	BD (*n* = 28)
Euthyroid	60 (68.2%)	16 (53.3%)	24 (80.0%)	20 (71.4%)
Subclinical Hypothyroidism	16 (18.2%)	10 (33.3%)	4 (13.3%)	2 (7.1%)
Overt Hypothyroidism	6 (6.8%)	4 (13.3%)	0 (0.0%)	2 (7.1%)
Subclinical hyperthyroidism	4 (4.5%)	0 (0.0%)	2 (6.7%)	2 (7.1%)
Overt hyperthyroidism	2 (2.3%)	0 (0.0%)	0 (0.0%)	2 (7.1%)

**Table 4:** Within-BD comparison according to mood stabilizer treatment.

Thyroid Parameter	Lithium Group, *n* = 20 (Mean ± SD)	Valproate Group, *n* = 8 (Mean ± SD)	*p*-Value
TSH	2.39 ± 1.64	2.59 ± 1.36	0.743
FT4	16.53 ± 2.20	14.01 ± 2.28	0.020
FT3	4.37 ± 0.81	4.46 ± 0.94	0.817

**Table 5:** Correlation matrix (r) between hormone levels and psychiatric scale scores.

Variable	TSH	FT4	FT3	MADRS	HAM-A	YMRS
TSH	1.00	−0.15	−0.22	0.45 *	0.38 *	−0.10
FT4	−0.15	1.00	0.68	−0.18	−0.12	0.30 *
FT3	−0.22	0.68	1.00	−0.25	−0.19	0.42 *
MADRS	0.45 *	−0.18	−0.25	1.00	0.55	0.10
HAM-A	0.38	−0.12	−0.19	0.55	1.00	0.05
YMRS	−0.10	0.30 *	0.42 *	0.10	0.05	1.00

* *p* < 0.05, statistically significant.

**Table 6:** Model Summary for the initial model.

Model	R	R^2^	Adjusted R^2^	Standard Error of the Estimate
Initial model	0.716	0.512	0.379	5.642

**Table 7:** ANOVA for the initial regression model.

Source	Sum of Squares	df	Mean Square	F	*p*-Value
Regression	734.108	6	122.351	3.844	0.009
Residual	700.192	22	31.827		
Total	1434.300	28			

**Table 8:** Regression coefficients for the initial model.

Predictor	B	SE	β	t	*p*	95% CI Lower	95% CI Upper	VIF
Constant	25.555	5.778		4.423	<0.001	13.572	37.538	
Hypothyroidism vs. euthyroidism	6.262	2.495	0.412	2.51	0.02	1.088	11.436	1.747
Hyperthyroidism vs. euthyroidism	5.374	4.828	0.194	1.113	0.278	−4.639	15.388	1.42
Age	−0.09	0.103	−0.133	−0.873	0.392	−0.304	0.124	7.926
Male sex	6.276	2.472	0.424	2.539	0.019	1.15	11.402	1.719
Episode duration	0.198	0.407	0.079	0.486	0.632	−0.646	1.041	5.885
Medication: anxiolytic + SSRI vs. dual-action antidepressant	1.704	2.719	0.108	0.627	0.537	−3.934	7.342	1.857

**Table 9:** Model Summary for the final model.

Model	R	R^2^	Adjusted R^2^	Standard Error of the Estimate
Final model	0.697	0.485	0.423	5.435

**Table 10:** ANOVA for the final regression model.

Source	Sum of Squares	df	Mean Square	F	*p*-Value
Regression	695.861	3	231.954	7.853	0.0007
Residual	738.439	25	29.538		
Total	1434.300	28			

**Table 11:** Regression coefficients for the final model.

Predictor	B	SE	β	t	*p*	95% CI Lower	95% CI Upper	VIF
Constant	23.274	1.375		16.925	<0.001	20.442	26.106	
Hypothyroidism vs. euthyroidism	6.036	2.342	0.397	2.577	0.016	1.212	10.859	1.413
Hyperthyroidism vs. euthyroidism	6.755	4.099	0.243	1.648	0.112	−1.687	15.196	1.074
Male sex	6.343	2.25	0.429	2.819	0.009	1.708	10.977	1.488

## Data Availability

The original contributions presented in this study are included in the article. Further inquiries can be directed to the corresponding authors.

## References

[1] Sonino N., Fava G.A., Aron D.C., Guidi J. (2025). The Role of Interviewing in Endocrine Practice. J. Endocrinol. Investig..

[2] Song X., Feng Y., Yi L., Zhong B., Li Y. (2023). Changes in Thyroid Function Levels in Female Patients with First-Episode Bipolar Disorder. Front. Psychiatry.

[3] Hossain M. (1970). Neurological and Psychiatric Manifestations in Idiopathic Hypoparathyroidism: Response to Treatment. J. Neurol. Neurosurg. Psychiatry.

[4] Lekurwale V., Acharya S., Shukla S., Kumar S. (2023). Neuropsychiatric Manifestations of Thyroid Diseases. Cureus.

[5] Fukao A., Takamatsu J., Arishima T., Tanaka M., Kawai T., Okamoto Y., Miyauchi A., Imagawa A. (2020). Graves’ Disease and Mental Disorders. J. Clin. Transl. Endocrinol..

[6] Smith S.M., Vale W.W. (2006). The Role of the Hypothalamic-Pituitary-Adrenal Axis in Neuroendocrine Responses to Stress. Dialogues Clin. Neurosci..

[7] Mincă A., Mincă D.I., Calinoiu A.L., Gheorghiță V., Popescu C.C., Rusu A., Cristea A.M., Mincă D.G. (2024). Myasthenia Gravis Triggered by a COVID-19 Infection: A Case Report and Literature Review. Cureus.

[8] McEwen B.S., Gray J.D., Nasca C. (2015). 60 YEARS OF NEUROENDOCRINOLOGY: Redefining Neuroendocrinology: Stress, Sex and Cognitive and Emotional Regulation. J. Endocrinol..

[9] Van Boxtel M.P.J., Menheere P.P.C.A., Bekers O., Hogervorst E., Jolles J. (2004). Thyroid Function, Depressed Mood, and Cognitive Performance in Older Individuals: The Maastricht Aging Study. Psychoneuroendocrinology.

[10] Shekhar S., Hall J.E., Klubo-Gwiezdzinska J. (2021). The Hypothalamic–Pituitary–Thyroid Axis and Sleep. Curr. Opin. Endocr. Metab. Res..

[11] Lazarus J.H. (2012). Thyroid Hormones and Cognitive Function. Expert. Rev. Endocrinol. Metab..

[12] Samuels M.H. (2014). Psychiatric and Cognitive Manifestations of Hypothyroidism. Curr. Opin. Endocrinol. Diabetes Obes..

[13] Hage M.P., Azar S.T. (2012). The Link between Thyroid Function and Depression. J. Thyroid. Res..

[14] Petersen P. (1968). Psychiatric Disorders in Primary Hyperparathyroidism. J. Clin. Endocrinol. Metab..

[15] Trifu S. (2019). Neuroendocrine Insights into Burnout Syndrome. Acta Endocrinol..

[16] Biondi B., Cappola A.R., Cooper D.S. (2019). Subclinical Hypothyroidism: A Review. JAMA.

[17] Levy M.J., Koulouri O., Gurnell M. (2013). How to Interpret Thyroid Function Tests. Clin. Med..

[18] Samuels M.H., Bernstein L.J. (2022). Brain Fog in Hypothyroidism: What Is It, How Is It Measured, and What Can Be Done About It. Thyroid.

[19] Wildisen L., Moutzouri E., Beglinger S., Syrogiannouli L., Cappola A.R., Åsvold B.O., Bakker S.J.L., Ceresini G., Dullaart R., Ferrucci L. (2019). Subclinical Thyroid Dysfunction and Depressive Symptoms: Protocol for a Systematic Review and Individual Participant Data Meta-Analysis of Prospective Cohort Studies. BMJ Open.

[20] Benseñor I.M., Nunes M.A., Sander Diniz M.D.F., Santos I.S., Brunoni A.R., Lotufo P.A. (2016). Subclinical Thyroid Dysfunction and Psychiatric Disorders: Cross-sectional Results from the Brazilian Study of Adult Health (ELSA-Brasil). Clin. Endocrinol..

[21] Pyun J.-M., Park Y.H., Kim S. (2022). Subclinical Hypothyroidism and Cognitive Impairment. JAD.

[22] National Guideline Centre (UK) (2019). Thyroid Function Tests: Thyroid Disease: Assessment and Management: Evidence Review C.

[23] Tang R., Wang J., Yang L., Ding X., Zhong Y., Pan J., Yang H., Mu L., Chen X., Chen Z. (2019). Subclinical Hypothyroidism and Depression: A Systematic Review and Meta-Analysis. Front. Endocrinol..

[24] Ma Y., Wang M., Zhang Z. (2024). The Association between Depression and Thyroid Function. Front. Endocrinol..

[25] Bode H., Ivens B., Bschor T., Schwarzer G., Henssler J., Baethge C. (2021). Association of Hypothyroidism and Clinical Depression: A Systematic Review and Meta-Analysis. JAMA Psychiatry.

[26] Liu S., Chen X., Li X., Tian L. (2024). Thyroid Hormone Levels in Patients with Bipolar Disorder: A Systematic Review and Meta-Analysis. BMC Endocr. Disord..

[27] Zhao S., Zhang X., Zhou Y., Xu H., Li Y., Chen Y., Zhang B., Sun X. (2021). Comparison of Thyroid Function in Different Emotional States of Drug-Naïve Patients with Bipolar Disorder. BMC Endocr. Disord..

[28] Norman S.J., Carney A.C., Algarin F., Witt B., Witzel I.M., Rodriguez P.M., Mohyeldin M. (2024). Thyroid Dysfunction and Bipolar Disorder: A Literature Review Integrating Neurochemical, Endocrine, and Genetic Perspectives. Cureus.

[29] Segarceanu L.-M., Zanfir A.-G., Minca D.G., Trifu S. (2025). Evaluation of Inflammatory Markers in Perception Disorders in Major Psychiatric Pathology. Int. J. Mol. Sci..

[30] Fan T., Luo X., Li X., Shen Y., Zhou J. (2024). The Association between Depression, Anxiety, and Thyroid Disease: A UK Biobank Prospective Cohort Study. Depress. Anxiety.

[31] Siegmann E.-M., Müller H.H.O., Luecke C., Philipsen A., Kornhuber J., Grömer T.W. (2018). Association of Depression and Anxiety Disorders With Autoimmune Thyroiditis: A Systematic Review and Meta-Analysis. JAMA Psychiatry.

[32] Tibaldi J.M., Surks M.I. (1985). Effects of Nonthyroidal Illness on Thyroid Function. Med. Clin. N. Am..

[33] Radhakrishnan R., Calvin S., Singh J.K., Thomas B., Srinivasan K. (2013). Thyroid Dysfunction in Major Psychiatric Disorders in a Hospital Based Sample. Indian J. Med. Res..

[34] Zhou J., Zhu L. (2024). Shared Genetic Links between Hypothyroidism and Psychiatric Disorders: Evidence from a Comprehensive Genetic Analysis. Front. Endocrinol..

[35] Feldman A.Z., Shrestha R.T., Hennessey J.V. (2013). Neuropsychiatric manifestations of thyroid disease. Endocrinol. Metab. Clin. N. Am..

[36] Fliers E., Boelen A. (2021). An Update on Non-Thyroidal Illness Syndrome. J. Endocrinol. Investig..

[37] Rozing M.P., Westendorp R.G.J., Maier A.B., Wijsman C.A., Frölich M., De Craen A.J.M., Van Heemst D. (2012). Serum Triiodothyronine Levels and Inflammatory Cytokine Production Capacity. Age.

[38] Małujło-Balcerska E., Pietras T. (2023). Deiodinase Types 1 and 3 and Proinflammatory Cytokine Values May Discriminate Depressive Disorder Patients from Healthy Controls. J. Clin. Med..

[39] Jucevičiūtė N., Žilaitienė B., Aniulienė R., Vanagienė V. (2019). The Link between Thyroid Autoimmunity, Depression and Bipolar Disorder. Open Med..

[40] Koc D., Ince E., San T., Akan P., Paketci A., Bober E., Tecirli N.D., Inal N., Akay A.P. (2022). Association between Thyroid Autoimmunity and Antidepressant Treatment-Emergent Mania in Pediatric Mood Disorders. Psychiatry Res..

[41] Jurado-Flores M., Warda F., Mooradian A. (2022). Pathophysiology and Clinical Features of Neuropsychiatric Manifestations of Thyroid Disease. J. Endocr. Soc..

[42] Keller J., Gomez R., Williams G., Lembke A., Lazzeroni L., Murphy G.M., Schatzberg A.F. (2017). HPA Axis in Major Depression: Cortisol, Clinical Symptomatology and Genetic Variation Predict Cognition. Mol. Psychiatry.

[43] Osimo E.F., Pillinger T., Rodriguez I.M., Khandaker G.M., Pariante C.M., Howes O.D. (2020). Inflammatory Markers in Depression: A Meta-Analysis of Mean Differences and Variability in 5,166 Patients and 5,083 Controls. Brain Behav. Immun..

[44] Min X., Wang G., Cui Y., Meng P., Hu X., Liu S., Wang Y. (2023). Association between Inflammatory Cytokines and Symptoms of Major Depressive Disorder in Adults. Front. Immunol..

[45] Benedetti F., Aggio V., Pratesi M.L., Greco G., Furlan R. (2020). Neuroinflammation in Bipolar Depression. Front. Psychiatry.

[46] Capatina T.-F., Oatu A., Babasan C., Trifu S. (2025). Translating Molecular Psychiatry: From Biomarkers to Personalized Therapies—A Narrative Review. Int. J. Mol. Sci..

[47] Guillen-Burgos H.F., Vanegas V., Gonzalez I., Caicedo A.M., Arango V., Galvez-Florez J.F., Anaya J.-M., McIntyre R.S., Terán W. (2025). Shared Genetic Architecture Among Severe Mental Disorders: A System Biology Approach Based on Protein-Protein Interaction. Brain Behav..

[48] Trifu A.S., Pietreanu A.C., Vasile A.I., Trifu S. (2025). Cognitive dysfunction in patients with major depressive disorder: An observational cross-sectional study in inpatients in a tertiary center in Romania. Balneo PRM Res. J..

[49] Touma K.T.B., Zoucha A.M., Scarff J.R. (2017). Liothyronine for Depression: A Review and Guidance for Safety Monitoring. Innov. Clin. Neurosci..

[50] Cooper-Kazaz R., Lerer B. (2008). Efficacy and safety of triiodothyronine supplementation in patients with major depressive disorder treated with specific serotonin reuptake inhibitors. Int. J. Neuropsychopharmacol..

[51] Walshaw P.D., Gyulai L., Bauer M.S., Calimlim B., Sugar C.A., Whybrow P.C. (2018). Adjunctive thyroid hormone treatment in rapid cycling bipolar disorder: A double-blind placebo-controlled trial of levothyroxine (L-T4) and triiodothyronine (T3). Bipolar Disord..

[52] Dickerman A.L., Barnhill J.W. (2012). Abnormal thyroid function tests in psychiatric patients: A red herring?. Am. J. Psychiatry..

[53] Premachandra B.N., Kabir M.A., Williams I.K. (2006). Low T3 syndrome in psychiatric depression. J. Endocrinol. Investig..

